# α1 Adrenergic Receptors Mediate Panic-like Defensive Behavior in Alcohol-Drinking but Not Alcohol-Naïve Rats

**DOI:** 10.3390/ph18040484

**Published:** 2025-03-28

**Authors:** Thatiane De Oliveira Sergio, Jacob Kellner, Sarah Wean, Frederic W. Hopf

**Affiliations:** 1 Department of Psychiatry, Indiana University School of Medicine (IUSOM), Indianapolis, IN 46202, USA; jpkellner316@gmail.com (J.K.); swean@iu.edu (S.W.);; 2 Stark Neuroscience Research Institute, IUSOM, Indianapolis, IN 46202, USA

**Keywords:** panic, anxiety, α1 adrenergic receptors, alcohol drinking

## Abstract

**Background:** Most animals display different defensive behavioral strategies during imminent or potential threats. These responses are relevant for understanding human behavioral disorders. In addition, α1 adrenergic receptors (α1ARs) are blocked by prazosin and regulate a diverse set of behaviors, including alcohol drinking related to anxiety in humans, alcohol intake in rats, responses to strong acute stresses (like restraint), and several forms of cognitive flexibility. However, the role of α1ARs in regulating panic-like escape behavior remains unexplored. **Methods:** Male and female Wistar rats were chronic alcohol drinkers and age-matched alcohol naïves. Animals received an injection of 0.75 mg/kg of prazosin or vehicle and then were exposed to the elevated T maze (ETM) to evaluate avoidance and escape behavior. One week later, animals underwent the light-dark test (LDT) and open field test. **Results:** α1AR inhibition with prazosin increased latency for escape in male and female alcohol drinkers, with no significant effects in alcohol-naïve controls. There were also interesting impacts from alcohol drinking, including a decrease in ETM avoidance in female but not male drinkers. In addition, prazosin increased latency to enter the dark in LDT in female drinkers and male naïves. Although prazosin also decreased the number of transitions in males, no differences were found in open-field locomotion. **Conclusions:** These results suggest that α1ARs mediate escape-like behavior in male and female alcohol drinkers, shedding light on a novel therapy for alcohol problems related to panic and anxiety.

## 1. Introduction

Most animals exhibit defensive behaviors, such as fleeing (escape) and avoidance, which are regulated by complex brain circuits in response to potential or imminent threats and facilitate survival. As such, it has been suggested that psychiatric disorders of emotion in humans are related to the persistence or disruption of these behaviors [1,2]. Anxiety, for example, can be defined as a feeling of anticipation about future threats, resulting in a state of high arousal and negative emotions [3]. In some individuals, these feelings become persistent and debilitating, resulting in a pathological state.

Noradrenaline is a crucial neuromodulator that regulates arousal, anxiety, and alcohol addiction [4,5]. Noradrenaline acts through different adrenergic receptors (ARs), including α1ARs, which can contribute significantly to several important behavioral states. For example, negative feelings and anxiety can drive problem alcohol drinking [6,7]. In this context, a large randomized clinical trial in heavy human alcohol drinkers showed that the α1AR antagonist prazosin reduced alcohol craving and anxiety symptoms, but primarily in those with greater anxiety during protracted abstinence [8]. Prazosin has also been used to treat post-traumatic stress disorder symptoms, including nightmares [9]. Additionally, α1ARs regulate cortical arousal while responding to some levels of challenge [10,11,12], congruent with the suggested importance of α1ARs for maintaining cognitive flexibility, especially under stress [11,13], in addition to ARs energizing behavior [14,15].

In agreement, pre-clinical studies show α1AR regulation of several anxiety-like behaviors related to drinking in outbred rodents and alcohol-preferring rats [16,17] (detailed in Discussion). Furthermore, we previously showed that systemic prazosin in male rats decreases compulsive alcohol drinking [18], when intake persists despite negative consequences, which can be a major problem in treating alcohol use disorders [6,19,20,21]; prazosin also reduces alcohol-only intake [18]. Further, in a recent study using a novel model of binge alcohol drinking, we found that a higher dose of prazosin decreases alcohol consumption in both male and female rats, while a lower dose only reduces female binge alcohol drinking. This suggests that female binge consumption is more sensitive to α1ARs, perhaps reflecting a novel sex-targeted therapy [22].

However, despite growing findings about α1AR regulation of anxiety-like behaviors and alcohol drinking in rodents, little is known thus far about how this receptor can regulate different defensive behavior strategies related to anxiety. In the presence of potential threats, animals can perform avoidance behavior, while imminent danger often elicits an escape response, and different brain circuits can regulate these behaviors [1,2]. Thus, here we investigated whether systemic administration of prazosin in male and female long-term alcohol drinkers or alcohol-naïve controls would affect inhibitory avoidance and escape responses performed on the elevated T-Maze (ETM). Indeed, escape behavior in ETM has been related to human panic attacks, with inhibitory avoidance acquisition associated with the psychopathology of generalized anxiety disorder [23]. The prazosin dose chosen here was based on studies from our lab [22], where 0.75 mg/kg but not 0.25–0.5 mg/kg prazosin impacts alcohol drinking moderately. In comparison, higher doses (1.5 mg/kg) give stronger suppression of drinking across most rats. Briefly, we found that prazosin did not impact avoidance behavior, but α1AR inhibition did increase the latency of escape in male and female long-term drinkers but not alcohol-naïve, control rats. Also, to further investigate the antagonist effect on different aspects of anxiety-like behavior, we evaluated prazosin impacts in a Light-Dark Test (LDT), as well as testing in an open-field test (to control for any prazosin impacts on locomotion).

## 2. Results

### 2.1. Experiment 1: Impact of the α1 Adrenergic Receptor Antagonist Prazosin on Avoidance and Escape Behavior in the Elevated T-Maze

**Figure 1:**
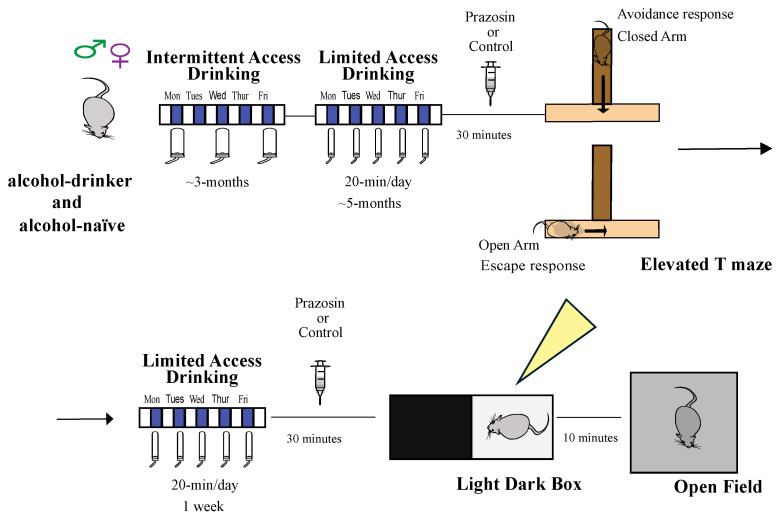
Experimental timeline. Drinking rats first had 3 months of Intermittent Access to two-bottle choice drinking of alcohol (IA2BC), then ~2 months of Limited Access Drinking (20 min/d 5 d/wk alcohol). Alcohol-naïve were age-matched (see Section 4). Behavior tests were at the same time when drinkers would normally have access to alcohol, but no alcohol was given before the Elevated T-Maze (ETM) or other tests. On ETM test day, rats were injected with 0.75 mg/kg prazosin or vehicle 30 min before ETM testing. After ETM testing, rats drank alcohol for 20 min that day and returned to Limited Access Drinking. One week after ETM, rats were injected with prazosin or vehicle (the same treatment, within-rat, as occurred during ETM) and then tested in the Light Dark box Task (LDT) and then 10 min later in open field. ETM, LDT, and open field details are in Section 4.

First, we investigated if systemic administration of prazosin (the α1AR antagonist) would impact the defensive behaviors of avoidance and escape performed on the ETM. Both sexes first drank alcohol under the Intermittent access to ethanol drinking paradigm for at least 3 months and were then switched to limited access drinking (5 days/week, 20 min/day) for at least 2 months. During the behavior test, animals were injected with prazosin or vehicle 30-min before being placed in the ETM (Figure 1). The alcohol-naïve control group was age-matched with the drinkers and kept in the same animal housing in the same conditions but not exposed to alcohol (see Section 4).

**Figure 2:**
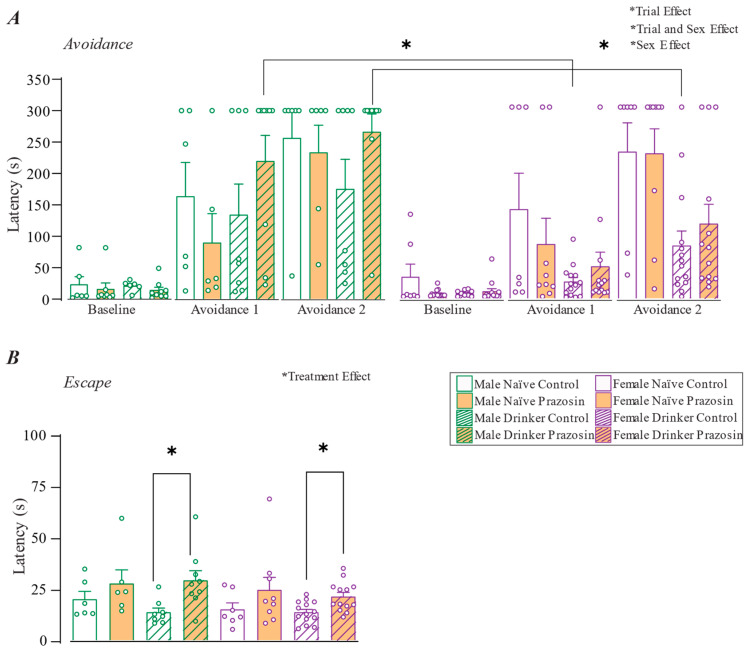
α1ARs regulate escape in ETM in alcohol drinkers but not alcohol-naive. (**A**) Latency to enter the open arms from the closed arm. Longer latency from baseline to avoidance tests 1 and 2 indicates learning to avoid entering the open arm, a critical first step to assure proper ETM behavioral responding. Learned avoidance, or lack thereof, is then typically compared during the Avoidance 2 trial. No significant differences looking at interactions between trial and drinking [F(2,116) = 2.953, *p =* 0.056], trial and treatment [F(2,116) = 0.872, *p =* 0.421], trial and sex and drinking [F(2,116) = 2.914, *p =* 0.058], trial and sex and treatment [F(2,116) = 0.032, *p =* 0.968], trial and drink and treatment [F(2,116) = 1.577, *p =* 0.211], and between trial and sex and drinking and treatment [F(2,116) = 0.721, *p =* 0.488]. (**B**) Latency to escape from the open arm to the closed arm. No interactions between sex and drinking [F(1,58) = 0.022, *p =* 0.883], sex and treatment [F(1,58) = 0.365, *p =* 0.548], drinking and treatment [F(1,58) = 0.134, *p =* 0.716], sex and drinking and treatment [F(1,58) = 1.003, *p =* 0.321]. Significant main effects and interactions are shown above each figure, with post-hocs indicating differences between a specific pair of conditions. *: *p* < 0.05.

We first examined Avoidance behavior (Figure 2A). Importantly, a three-way ANOVA with repeated measures shows a trial effect, suggesting that rats had successfully learned the inhibitory avoidance [F(2,116) = 34.828, *p <* 0.001]. In avoidance trial 1, the rat leaves the closed arm and enters the open arm, in theory, because they do not yet know that they are entering a threatening area. The longer latencies in avoidance trials 2 and 3 show that the rat has learned to not leave the closed arm because if it does, it will enter the open-arm areas (which they had “learned to fear and avoid” during the initial 30 min period when trapped in the open arm). This is a critical first step to assure interpretable performance in the ETM (see Section 3).

**Figure 3:**
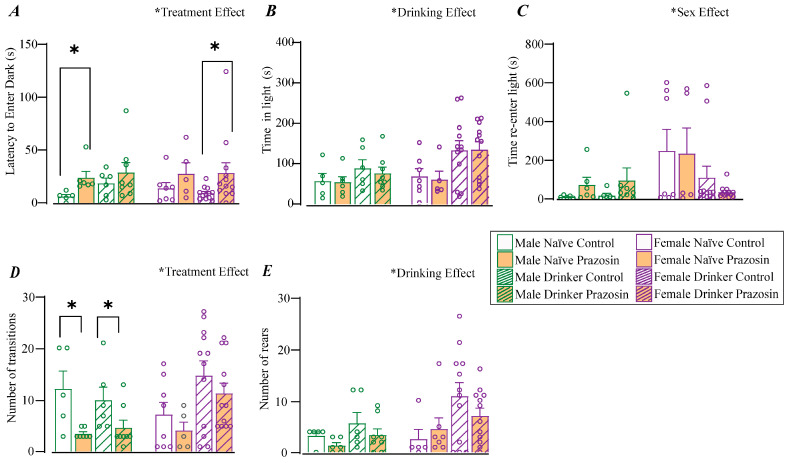
LDT behaviors. Rats are first placed on the lit side, facing away from the opening to the dark chamber. (**A**) Latency to first enter the dark chamber. For latency to first enter the dark chamber, there were no interactions between factors. [Sex and Drinking: F(1,61) = 1.190, *p* = 0.280; Sex and Treatment: F(1,61) = 0.008, *p* = 0.928; Drinking and Treatment: F(1,61) = 0.053, *p* = 0.819; Sex and Drinking and Treatment: F(1,61) = 0.202, *p* = 0.655]. (**B**) Total time in the lit chamber. No interactions for [Sex and Drinking: F(1,61) = 1.117, *p* = 0.295; Sex and Treatment: F(1,61) = 0.015, *p* = 0.903; Drinking and Treatment: F(1,61) = 0.181, *p* = 0.672; Sex and Drinking and Treatment: F(1,61) = 0.037, *p* = 0.849]. (**C**) Time to first re-enter the lit chamber after (**A**). No interactions for [Sex and Drinking: F(1,61) = 3.657, *p* = 0.061; Sex and Treatment: F(1,61) = 1.388, *p* = 0.244; Drinking and Treatment: F(1,61) = 0.042, *p* = 0.838; Sex and Drinking and Treatment: F(1,61) = 0.137, *p* = 0.713]. (**D**) Number of transitions between chambers. A three-way ANOVA showed no interactions for number of transitions [Sex and Drinking: F(1,61) = 3.888, *p* = 0.054; Sex and Treatment: F(1,61) = 0.695, *p* = 0.408; Drinking and Treatment: F(1,61) = 0.231, *p* = 0.633; Sex and Drinking and Treatment: F(1,61) = 0.165, *p* = 0.686]. (**E**) Number of rears. Significant main effects and interactions are shown above each figure, with post-hocs indicating differences between a specific pair of conditions. * *p* < 0.05.

We then examined whether alcohol drinking and/or α1AR inhibition impacted the expression of learned avoidance (avoidance trials 2 and 3). The overall ANOVA found an interaction between trial and sex [F(4,116) = 2.613, *p* = 0.039], but there were no interactions between other factors (most statistics for non-significant results are shown in the Figure 2). However, even though there was no main effect of treatment [F(1,58) = 0.054, *p* = 0.818], there were main effects of sex [F(2,58) = 4.816, *p* = 0.012] and drinking [F(1,58) = 4.303, *p* = 0.043]. In particular, alcohol drinking led females to express significantly less avoidance, expressed as entering the open arm sooner (a shorter latency to enter) in avoidance trial 3 in female drinkers. Otherwise, there were few post-hoc differences, except between the male drinker prazosin group and the female drinker control group for avoidance 1 (*p* = 0.012) and 2 (*p* = 0.019). Together, these findings suggest that alcohol drinking led females to express significantly less avoidance of the threatening open arm (even though the level of escape when placed in the open arm did not change, Figure 3), and, while speculative, decreased avoidance could relate to greater drinking problems observed in heavy-drinking women when compared to men [24]. However, the limited effects of prazosin suggested that α1ARs largely did not regulate these avoidance behaviors in the ETM.

For analysis of escape behavior, rats have three trials when they are placed in the same open arm they were confined to earlier in the training. The latency to escape is assessed when the rat enters the closed arm (having escaped from the threatening open arm). The latency is generally similar across the 3 escape trials, and the latencies for the three escape trials were averaged within each animal (see Section 4).

Escape showed a strongly significant effect of treatment [F(1,74) = 12.387, *p* < 0.001], but no main effect of sex [F(2,58) = 1.181, *p* = 0.314] or drinking [F(1,58) = 0.972, *p* = 0.328] (Figure 2B). Further, the three-way ANOVA showed no interaction between many different factors (see Figure 2), when post-hoc tests showed a significant difference between prazosin and vehicle for male drinkers (*p* = 0.011) and female drinkers (*p* = 0.004), but not for male or female alcohol-naïve rats. In particular, even though the level of escape behavior was similar in alcohol-drinkers and alcohol-naives, alcohol drinkers of both sexes seem to have undergone a neuro-adaptation such that α1ARs are now critical for sustaining the panic-like escape responses, while naïve escape was not regulated by α1ARs (Figure 2B). As a result, we speculate that α1ARs might contribute to panic-like aspects of mood disruption in heavy drinkers, leading to the clinical efficacy in this subset of human drinkers [8].

### 2.2. Experiment 2: α1AR Antagonist Effects in the Light-Dark Box Task (LDT)

One week after ETM, rats received an injection of prazosin or vehicle and, 30 min later, were placed on the brightly lit side of the LDT (Figure 1, see Section 4). The LDT involves two chambers, one lit and one dark, with a small opening between them, and assesses the conflict between aversion to light and the drive to explore [25,26]. As such, one set of LDT measures is more often related to aversion to light and subsequent anxiety-like behavior (latency to first enter the dark chamber, time in the lit chamber, and time to reenter the lit chamber) [25,26].

Latency to first enter the dark chamber is of primary interest, since it reflects the first movement from the novel, lit chamber into the dark chamber, perhaps reflecting conflict between the need to assess the novel situation and aversion to light. This measure showed a significant treatment effect [F(1,61) = 6.744, *p* = 0.012] (Figure 3A) but no main sex effect [F(1,61) = 0.003, *p* = 0.958] or drinking effect [F(1,61) = 0.212, *p* = 0.647] or interactions (see Figure 3). However, when comparing pairs of conditions (drinking-vs-naïve within sex but across different drug treatments), we found a difference between male naïve control and male naïve prazosin (*p =* 0.004) and between female drinker control and female drinker prazosin (*p =* 0.015), with no differences between other groups. Thus, these results suggest a more complex pattern, where α1ARs regulated this aspect of LDT anxiety-like behavior in male naïves and female drinkers, indicating that putative α1AR neuroadaptations with drinking (which impacted escape, Figure 2) also regulated other aspects of female but not male anxiety-like behavior.

Total time spent in the lit chamber is also an important and easier to interpret metric for anxiety-like behavior. However, a different pattern was observed when analyzing total time in the lit chamber (versus first entering the dark chamber). There was a main effect of drinking [F(1,61) = 5.154, *p* = 0.027] (Figure 3B), but not sex [F(1,61) = 1.903, *p* = 0.173] or treatment [F(1,61) = 2.507, *p* = 0.113], no interactions with three-way ANOVA (see Figure 3), and no significant post-hocs between groups. A third measure, the first time to re-enter the lit chamber (after moving from dark to lit in Figure 3A), is also of interest as a possible indicator of time spent first exploring the ostensibly more comfortable dark chamber. However, for this metric, there was a main effect of sex [F(1,61) = 5.325, *p* = 0.025] (Figure 3C) but not drinking [F(1,61) = 2.545, *p* = 0.117] or treatment [F(1,61) = 0.087, *p* = 0.769], no interactions (see Figure 3), and no post-hoc differences between groups. Thus, for time in light and time to reenter the light, present studies showed no effects of prazosin and some limited other differences (although not large enough effects to pass a post-hoc test).

Our LDT findings thus far suggest that prazosin and alcohol-drinking history have only limited effects on these aspects of anxiety-like behavior. Several other LDT measures have been considered more to indicate locomotor activity (see Section 3), including the number of transitions between the two chambers (Figure 3D) and the number of rears (Figure 3E). Interestingly, the number of transitions showed a main effect of treatment [F(1,61) = 8.340, *p* = 0.006] (Figure 3D), but sex [F(1,61) = 1.041, *p* = 0.312] or drinking [F(1,61) = 2.790, *p* = 0.101], and no interactions (see Figure 3). When comparing the paired conditions post hoc, prazosin decreased the number of transitions in both alcohol-naïve males (*p* = 0.043) and male drinkers (*p* = 0.032), with no effects in females. Thus, these findings perhaps suggest a more basic sex difference in how α1ARs mediate male but not female locomotor activity in the LDT, regardless of drinking status. However, as we will see below, prazosin inhibition of α1ARs did not regulate open field locomotion (Table 1). Thus, our results suggest the interesting possibility that males and females have a similar number of transitions in LDT, but that α1ARs are critical for sustaining such transitions only in males. This also suggests that the number of transitions in LDT captures some aspects of engagement that are different from locomotion per se, a direction for future studies.

Rearing is considered a basic defensive assessment behavior, although the mechanistic basis of rearing, relative to other anxiety- and panic-associated behavioral events, remains unclear. Here, rearing showed a main effect of drinking [F(1,61) = 6.563, *p* = 0.013], although with no differences between groups post-hoc. Further, there were no interactions [Sex and Drinking: F(1,61) = 1.163, *p* = 0.286; Sex and Treatment: F(1,61) = 0.042, *p* = 0.838; Drinking and Treatment: F(1,61) = 0.096, *p* = 0.757; Sex and Drinking and Treatment: F(1,61) = 0.029, *p* = 0.865], and no main effect of sex [F(1,61) = 3.789, *p* = 0.057] or treatment [F(1,61) = 2.476, *p* = 0.122]. Thus, our findings do not support alcohol, sex, or α1AR effects on rearing in the LDT. However, they do support the speculation that rearing can be mechanistically dissociated from other putative anxiety-related behaviors, at least under the conditions studied.

### 2.3. Experiment 3: α1AR Antagonist Prazosin in Open Field Test

Studies above suggest the possibility that α1ARs and alcohol-drinking history can alter avoidance, panic, and anxiety-related behaviors. However, changes in behavior could reflect more basic impacts on locomotion. Thus, animals were exposed to a 10 min open-field test (10 after LDT, Figure 1), and we analyzed the distance traveled and velocity. Prazosin did not affect the distance or velocity of animals (Table 1), and there was no main effect of drinking [F(1,56) = 0.754, *p =* 0.390] or treatment [F(1,56) = 0.014, *p =* 0.905]. There was a main effect of sex [F(1,56) = 6.468, *p =* 0.014], but no post-hoc differences between groups. Further, the three-way ANOVA showed no interaction between the factors for the distance traveled [Sex and Drinking: F(1,56) = 0.478, *p =* 0.493; Sex and Treatment: F(1,56) = 0.113, *p =* 0.738; Drinking and Treatment: F(1,56) = 1.669, *p =* 0.203; Sex and Drinking and Treatment: [F(1,56) = 0.647, *p =* 0.425].

For velocity, there were no main effects of drinking [F(1,56) = 1.074, *p =* 0.305], treatment [F(1,56) = 0.178, *p =* 0.675], or sex [F(1,48) = 2.147, *p* = 0.149], and no interactions between factors [Sex and Drinking: F(1,56) = 0.430, *p* = 0.515; Sex and Treatment: F(1,56) = 0.012, *p* = 0.913; Drinking and Treatment: F(1,56) = 0.283, *p* = 0.597; Sex and Drinking and Treatment: [F(1,56) = 0.927, *p* = 0.340]. Thus, it is unlikely that the effects reported above were simply the consequence of differential regulation of motor activity.

## 3. Discussion

Animals (including humans) can exhibit defensive behaviors such as avoidance and escape when faced with potential or imminent threats, respectively. Importantly, these behaviors have been associated with different human psychopathologies, with avoidance related to anxiety-like and escape with panic-like behaviors [1,2]. In addition, α1ARs have been linked to several aspects of responding under challenge, perhaps providing cognitive flexibility under stress, which can become maladaptive. However, it remains poorly understood whether any aspects of defensive and related behavior are regulated or not by α1ARs. Thus, here we examined α1ARs modulation through injection of prazosin, and we assessed avoidance and escape behaviors in male and female long-term alcohol-drinking rats. Most interesting, we found that escape behavior was regulated by α1ARs in alcohol drinkers but not in alcohol-naïve, age-matched control rats, suggesting an alcohol-related neuro-adaptation in the receptor mechanisms that regulate escape behavior. Other changes related to alcohol (but not α1AR) and sex differences are elaborated below.

In particular, the ETM is considered an ethological model that analyzes inhibitory avoidance and escape responses in the same animal (for review, see [23]). Pharmacological studies show that drugs used clinically to treat anxiety disorders, such as buspirone or benzodiazepines, decrease the latency of inhibitory avoidance, indicating that animals leave from the closed into the threatening open arm sooner with such treatments. In addition, drugs used to treat panic disorder impact escape behavior in ETM, but not avoidance, increasing the time before rats leave (escape from) the open arm. To further relate prazosin effects to anxiety-like behavior and/or locomotion, we also used the LDT and open field test.

As noted above, one notable finding was that α1AR inhibition reduced escape behavior (increased time in the open arm) in alcohol drinkers but not alcohol-naïve controls, and in both females and males. This was the case even with similar levels of escape behavior across vehicle conditions in drinkers and alcohol-naives. Thus, our findings interestingly suggest that drinkers express similar avoidance as alcohol-naïve, but some neuro-adaptation has occurred such that escape behavior is now regulated by α1ARs. While the “reason” for such a change is unclear, it is interesting that α1AR inhibition with prazosin in heavy-drinking humans reduces alcohol craving and mood disruptions, but with a particular impact in those with greater anxiety symptoms across longer-term withdrawal [8]. Thus, one possible speculation is that panic-like states could play a role in the drive for alcohol, at least in some heavy drinkers.

We found interesting but contrasting results when we examined inhibitory avoidance behavior. While prazosin did not impact avoidance, female alcohol drinkers had a significant decrease in the latency to avoidance, which suggests less avoidance and perhaps an anxiolytic-like effect of long-term alcohol drinking in females, at least under the conditions studied here. Importantly, despite the still higher prevalence of AUD in men, recent epidemiological studies show that alcohol use in women, and associated negative outcomes, have risen considerably during the past years [24]. One major concern is that women are more vulnerable to the harmful effects of excessive alcohol use. For example, women report higher rates of comorbid psychiatric conditions such as anxiety and mood disorders, and relapse in women is often related to heavy drinking as a maladaptive strategy to alleviate negative symptoms (drinking to cope) [27]. In contrast, men more often report heavy drinking and relapse in response to positive emotions and social factors [28]. Thus, the reduced avoidance in female drinkers in the ETM might reflect a more overall disruption of affect regulation, consistent with female rodents often showing greater compulsion-like alcohol drinking than males [29,30,31], and where heavy-drinking women can have more alcohol problems than men [24].

It was also interesting that α1ARs regulated some aspects of male LDT in both alcohol drinkers and controls, with no effect in females. In particular, α1ARs regulated the number of transitions between the two chambers (lit, dark) in males, with limited effects in any other LDT measure. While this number of transitions measured in LDT has often been considered perhaps an internal motor control, α1AR inhibition with prazosin had no impact on locomotor measures in the open field. Thus, one interesting speculation is that α1ARs specifically control specific aspects of males moving from threatening to more safe contexts (perhaps escaping from the freezing effects of light in order to escape into the darkness), although the converse (α1ARs regulating males entering into threatening lit conditions) is also possible. One way to address such questions would be to use autonomic measures such as heart rate variability to better assess subjective state. We previously used such methods to relate arousal state to alcohol drinking and anxiety in female and male alcohol-drinking rats [32,33]. Thus, future studies can better assess what specific aspects of emotion- and motivation-related responding are regulated by α1ARs in drinkers and non-drinkers and in males and females.

The observed α1ARs regulation of panic-related escape in ETM in alcohol drinkers, but not in alcohol-naïve controls (Figure 2A), is interesting in light of previous studies. More generally, the noradrenergic system is broadly linked to the modulation of defensive behaviors related to anxiety and alcohol drinking [16,17,34]. Importantly, other studies specifically link α1ARs to some aspects of panic-like behavior. In rats, α1AR density is diminished by sodium lactate, a substance that can trigger panic in humans who suffer panic attacks. Furthermore, α1AR inhibition in the dorsal raphe nucleus or medial hypothalamus decreases escape behavior (running and jumping) induced by local block of GABA_A_ receptors. Further, lesioning the Locus Coeruleus, a main source of brain noradrenaline, decreases the duration of the freezing during an unconditioned aversive stimulus [35]. Also, in agreement with our results, enhancing α1AR signaling through genetic manipulation or pharmacology showed no effect in LDT or in the elevated plus maze [36], although prazosin (0.75 mg/kg; the same dose used here) decreased anxiety-like behavior on the marble-burying test in male and female mice exposed to chronic stress and alcohol drinking [17]. Based on our results and these findings, we speculate that α1ARs are not largely required for basal situations lacking stressors and potential threats, but that α1ARs can play an important role during responses in the presence of imminent threat in both male and female rodents. In addition, future studies are needed to understand how α1ARs impact responding to more salient threats such as looming disks and shock probes in the bedding.

It is also particularly interesting here that α1ARs regulated escape behavior in both sexes in alcohol drinkers but not in naives. Thus, alcohol-drinking can induce neuroadaptations in α1ARs such that these receptors now regulate escape, while they played no role in escape in alcohol-naïve rats. Thus, α1ARs have the potential to be potent therapeutic agents in humans [8,9] and might be persistently recruited under pathological conditions. Thus, interesting future studies could examine whether chronic stress or other protracted challenges induce similar α1AR neuroadaptations as alcohol here.

The α1ARs are of clear interest as a therapeutic target for alcohol based on rodent studies (reviewed in [21]), and in particular where the α1AR blocker prazosin reduces alcohol-related states in heavy human drinkers that have greater mood disruptions during protracted withdrawal [8]. However, it is important to note that prazosin and related compounds reduce blood pressure, and studies in human drinkers have noted standing hypotension [37] and more drowsiness and edema [38]. These are significant side effects when considering use for therapy and require physician monitoring to be safe. However, we find that prazosin is effective at reducing female binge drinking at lower doses of prazosin, while higher prazosin doses reduce alcohol intake in both sexes [22]. Thus, as we’ve argued, lower doses may reflect effective therapy in humans while minimizing potential side effects.

Prior results from our lab showed that prazosin (0.3 μg) directly in the anterior insula cortex, a brain area related to negative salience and alcohol compulsion, reduces both regular and compulsion-like alcohol drinking [18]. However, this same dose in the medial prefrontal cortex does not affect alcohol consumption [39], although previous studies linked α1ARs in this cortical area to cognitive flexibility [11,12,13]. Future studies will need to evaluate the brain circuitries related to α1ARs modulation on escape responses and other anxiety-related behaviors in long-term drinkers and non-drinkers.

Previous studies have examined changes in anxiety- and fear-related behavior in female and male rodents after a history of protracted alcohol drinking. Other studies using longer-term (≥8 wk) intermittent access drinking find increased anxiety-like behavior in males (e.g., [40] in elevated plus maze, avoidance of open, elevated platforms) and marble burying, with no motor changes. Studies with shorter intake history (≤6 wk) find mixed results, with no drinking-related anxiety-like behavior changes in either sex [41], female-only changes [42], or male differences in elevated plus maze and female in another task (avoiding predator context) [43]. Thus, further studies are needed to better understand how alcohol drinking alters the expression of the many aspects of anxiety and defensive behaviors.

Taken together, our results highlight the critical role of α1ARs for modulating panic-like responses in both sexes of long-term alcohol drinkers, but not alcohol-naives. In addition, long-term drinking impacted avoidance behavior in females but not males. Thus, our findings suggest crucial sex-similar and sex-different neuroadaptations in defensive behavior circuitry, shedding light on future studies to investigate the specific brain pathways related to these behaviors, as well as novel therapy for alcohol problems. 

## 4. Methods

### 4.1. Animals

The Indiana University Institutional Animal Care and Use Committee (IACUC) approved all animal care and handling procedures (protocol number 22097, approved on 1 December 2022), following the National Institutes of Health guidelines. Male and female Wistar rats arrived on PN45-50 and were single-housed in clear plastic cages, with the same number of males and females kept in animal housing under a 12-h light/dark (dark phase between 11:30 a.m. and 11:30 p.m.), with food and water *ad libitum*. All behavioral sessions were performed during the dark phase of the cycle under red lights.

### 4.2. Drinking Methods

Long-term alcohol drinking started after ~2 wk of acclimatization to animal housing. Rats designated for the drinking group began alcohol exposure under intermittent-access two-bottle-choice (20% alcohol in water vs. water-only, IA2BC), with ~24-h intake/day starting ~1 h into the dark cycle on Monday, Wednesday, and Friday, as described in [21,44] (Figure 1). After >3 mo IA2BC, rats switched to 20 min/d, 5 d/wk drinking for ~2 mo [30,39,45]. Naïve groups were aged matched with drinkers and kept in the same animal housing, with the presence of one water bottle.

### 4.3. Drugs

Prazosin hydrochloride, from Sigma-Aldrich (Indianapolis, IN, USA), was diluted in water and injected i.p. 30-min before behavioral sessions. The dose of 0.75 mg/kg was chosen based on our previous studies, showing prazosin’s effect on compulsion-like and binge alcohol drinking [18,22].

### 4.4. Behavioral Sessions

Before all behavioral tests, animals were handled for at least one week for ~5 min/day. The researcher responsible for conducting experiments was blinded for treatment received by animals during behavioral sessions and for video analysis. Also, males and females were tested on separate days.

All behavior tests occurred at the same time of day when drinkers would normally have access to alcohol but were tested before alcohol intake. The animals were randomly assigned for pharmacological treatment (prazosin or control), and we made sure that the number of animals that received drug or control was balanced between alcohol drinkers and non-drinkers.

### 4.5. Elevated T-Maze

The ETM apparatus was made of black acrylic with three arms of equal dimensions (50 × 12 cm) [46,47]. One arm was enclosed by 40 cm high walls and perpendicular to two opposed open arms, making a “T”. The whole apparatus was elevated 50 cm above the floor, and to avoid falls, open arms were surrounded by a 1 cm high clear acrylic wall.

ETM followed previous methods [48]. To enhance escape response, one day before behavioral testing, rats were exposed to one open arm for 30 min. For this, an acrylic barrier isolated this open arm from the rest of the maze. On the test day (the next day), a balanced number of alcohol drinkers or naïve animals of the same sex were randomly allocated to treatment groups and received a systemic injection of prazosin 0.75 mg/kg or vehicle (water). 30 min later, animals were tested in ETM. The test was performed as we described before [46] and was initiated by measuring inhibitory avoidance. Rats were first placed at the distal end of the closed arm, facing the intersection of the arms. The time taken by animals to leave the closed arm into the open arms with all four paws was recorded (baseline latency). The same assessment was repeated in two subsequent trials (avoidance 1 and 2) using 30-s inter-trial intervals.

The ETM is based on rodents innate fear of open places and heights, where being exposed to open arms is an aversive experience. Thus, repeatedly placing rats inside the enclosed arm and allowing them to explore the maze allows animals to learn inhibitory avoidance (for review, see [23]). 30 s following avoidance trial 2, escape trials were carried out. Rats were placed at the end of the same open arm they were in for the 30-min exposure, and the latency to enter the closed arm with four paws was recorded three consecutive times (escape 1, 2, and 3), again with 30-s inter-trial intervals. When the rat is placed at the end of the open arm, it moves towards the closed arm, presumably performing an escape response. The latency to leave the open arm does not usually change with consecutive trials, and thus escape trails are averaged (see [23]). A cut-off time of 300 s was established for avoidance and escape latencies. Immediately after the escape measurement, rats returned to animal housing and were exposed to alcohol bottles for 20 min. Escape results were averaged from the three escape trials, as before [49].

### 4.6. Light Dark Box Test (LDT)

LDT was performed in an acrylic box with two equal-sized chambers (41 × 41 × 41 cm), one black (with a black lid) and one white (with 100 lux light and a clear lid), connected by an 8 × 8 cm open doorway between chambers [50] LDT tests were performed one week after ETM, and each rat received the same treatment (prazosin or vehicle) in LDT as in ETM (Figure 3). We lost some animals due to technical problems with video recording (2 males, one drinker and one naïve, and 4 females, 3 drinkers and 1 naive). Also, some animals did not re-enter the light and were excluded from analysis (2 males drinker vehicle and 1 drinker prazosin; 5 females: 3 naïve prazosin, 1 drinker vehicle, and 1 drinker prazosin). The test began 30 min after systemic injection of prazosin or vehicle, with the animal being placed in the lit compartment facing away from the doorway and allowed to explore for 10 min. We analyzed the latency to first move into the dark chamber, the latency to re-enter the light chamber, and the total time in the light chamber. In addition, we analyzed the number of transitions and rearings, measures more related to locomotion and exploration.

### 4.7. Open Field

After LDT, rats returned to their home cage for 5-min and then were placed in the Open Field apparatus (acrylic square arena 60 × 60 cm, illuminated by red light). Rats were placed in the center of the arena and recorded for 10 min. Total distance and average velocity were determined using EthoVision XT10 (Noldus, Wageningen, The Netherlands). We lost 6 animals due to technical problems with camera recording and software analysis (1 male drinker prazosin, 2 female naïve vehicles, 1 female naïve prazosin, 1 female drinker vehicle, and 1 female drinker prazosin).

### 4.8. Statistics

Three-way ANOVA with repeated measures was used for all analyses, with sex, drinking condition, and treatment as independent factors. When appropriate, post-hoc comparisons were performed using *t*-tests or multiple comparisons. All data are shown as mean ± standard error of the mean. However, we note that some of our sample sizes are low (such as 6 alcohol-naïve, control males), and thus negative results should be treated with some caution.

## Figures and Tables

**Table 1:** Effect (mean ± S.E.M.) of control or prazosin injection on distance traveled and velocity of male and female alcohol drinkers or naïve rats in the open-field test.

Sex	Groups	Distance	Velocity
	Naïve Control	1806.3 ± 213.3	10.2 ± 1.9
Males	Naïve Prazosin	1821.7 ± 441.3	10.1 ± 3.1
	Drinker Control	1960.9 ± 724.7	7.6 ± 10.1
	Drinker Prazosin	1491.9 ± 220.4	8.5 ± 1.1
	Naïve Control	2845.7 ± 589.1	6.2 ± 1.1
Females	Naïve Prazosin	3993.7 ± 1509.5	8.8 ± 3.3
	Drinker Control	3134.0 ± 781.9	7.6 ± 1.6
	Drinker Prazosin	2179.0 ± 414.8	6.4 ± 0.7

## Data Availability

The original contributions presented in the study are included in the article, further inquiries can be directed to the corresponding author.
